# Sexual conflict, heterochrony and tissue specificity as evolutionary problems of adaptive plasticity in development

**DOI:** 10.1098/rspb.2023.1854

**Published:** 2023-10-11

**Authors:** Asher D. Cutter

**Affiliations:** Department of Ecology and Evolutionary Biology, University of Toronto, Toronto, Ontario, Canada M5S 3B2

**Keywords:** sexual conflict, adaptive plasticity, tissue specificity, heterochrony, negative pleiotropy, gene regulation

## Abstract

Differential gene expression represents a fundamental cause and manifestation of phenotypic plasticity. Adaptive phenotypic plasticity in gene expression as a trait evolves when alleles that mediate gene regulation serve to increase organismal fitness by improving the alignment of variation in gene expression with variation in circumstances. Among the diverse circumstances that a gene encounters are distinct cell types, developmental stages and sexes, as well as an organism's extrinsic ecological environments. Consequently, adaptive phenotypic plasticity provides a common framework to consider diverse evolutionary problems by considering the shared implications of alleles that produce context-dependent gene expression. From this perspective, adaptive plasticity represents an evolutionary resolution to conflicts of interest that arise from any negatively pleiotropic effects of expression of a gene across ontogeny, among tissues, between the sexes, or across extrinsic environments. This view highlights shared properties within the general relation of fitness, trait expression and context that may nonetheless differ substantively in the grain of selection within and among generations to influence the likelihood of adaptive plasticity as an evolutionary response. Research programmes that historically have focused on these separate issues may use the insights from one another by recognizing their shared dependence on context-dependent gene regulatory evolution.

## 1. Introduction

**Figure 1.**
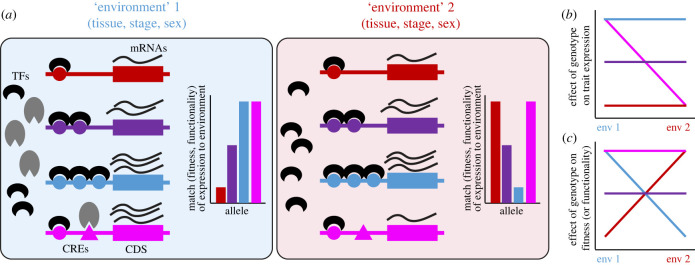
Gene regulation through different *cis*-acting alleles can mediate adaptive plasticity in gene expression. (*a*) Allelic differences in gene regulation can alter the amount of constitutive expression across contexts (red/purple/blue alleles conferring constitutive low/medium/high expression) or plastically alter the amount of expression in different contexts (pink allele). Matching of allelic expression to a given context affects the realized fitness or functionality in that ‘environmental’ context. The environmental context may represent extrinsic environments, as considered in classic plasticity conceptions, or the context of expression may correspond to distinct tissues, ontogenetic stages, or sexes. Note that two ‘environments’ are depicted for brevity, but plasticity is best contextualized with respect to many conditions [1]. (*b*) Norm of reaction for alleles conferring plastic and non-plastic trait expression differences. (*c*) Environmental tolerance curves projects the relationship between fitness (or functionality) and environmental context from the more general relationship between fitness, phenotype, and environment from which reaction norms are an alternate projection [2]. Curve colours in (*b*) and (*c*) correspond to the alternative alleles represented in (*a*). TF, transcription factor; CRE, *cis*-regulatory element; CDS, gene coding sequence.

All organisms face the challenge of expressing the right genes at the right time and place to promote their survival and reproduction. The molecular answer to this challenge is, of course, conditional regulation of gene expression in the control of trait development (figure 1) [3]. Differential expression over time and space is ubiquitous within and among organisms [4]. Even in the absence of structural changes to the protein sequences encoded by genes, mutation to regulatory elements provides the kind of change that can give rise to specialized tissues over the developmental timecourse of ontogeny, that can produce sexually dimorphic traits, or that can lead to environment-dependent trait states. Differential expression of genes thus resolves some of the constraints on all cells of an organism and all individuals of a species that must share the same set of instructions encoded by the genome. This well-established feature of molecular developmental biology—dynamic gene regulation—represents one of the most fundamental manifestations of phenotypic plasticity [5,6]. Here, I will explore how adaptive plasticity of gene expression provides a convenient and insightful framework to view, and translate among, a diversity of evolutionary problems in the resolution of evolutionary conflicts that involve negatively pleiotropic effects of gene expression.

Phenotypic plasticity describes the ability of a single genotype to produce multiple context-dependent phenotypes [1,7–9]. When such trait variation aligns with fitness, then the trait variation represents adaptive phenotypic plasticity, as opposed to trait variation as phenotypic noise or stress-induced dysfunction that is non-adaptive or even maladaptive [10]. Adaptive plasticity allows a single genotype of an organism to confer high fitness under a range of circumstances, and so is sometimes framed in terms of robustness of fitness to perturbation [11,12]. This kind of adaptive response differs from local adaptation or ecological specialization in which different genotypes confer high fitness under distinct circumstances [13].

**Figure 2.**
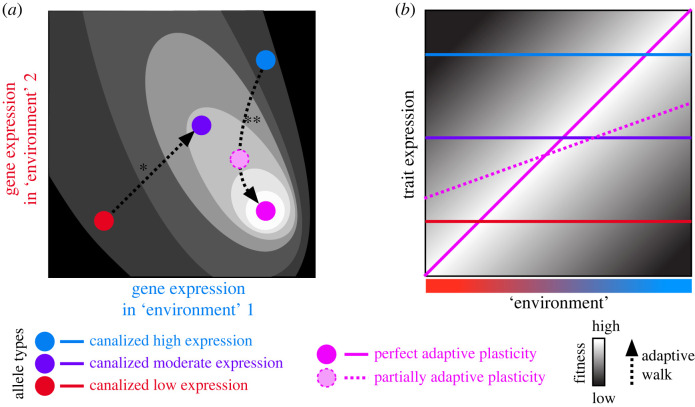
Fitness landscape with respect to gene expression traits in different contexts. (*a*) Evolution of non-plastic high (blue) or low (red) expression could follow an adaptive walk (*) to intermediate constitutive expression (purple) that balances the trade-off of fitness costs and benefits across expression contexts. Evolution of plastic context-dependent expression (pink) can allow genotypes to trace adaptive walks (**) to fitness peaks that are inaccessible to genotypes with constitutive expression of canalized traits. (*b*) General relationship of the fitness landscape that depends on both trait expression and environmental context [2]. Genotypes that confer non-plastic expression correspond to horizontal slices through fitness-environment space, whereas genotypes conferring plastic expression can trace the fitness ridge.

Research on adaptive plasticity most frequently is associated with phenotypic differences among organisms that arise in response to distinct cues of the external environment [9,14]. This relationship typically gets characterized as the reaction norm of phenotype as a function of environment, or more generally by the joint relationship between fitness, phenotype and environmental context (figure 2) [2]. The principle, however, applies to any trait and any cue. Gene expression, as one of the most basic traits that an organism can have, and the distinct ‘environments’ that a gene experiences all are conducive to analysis from the perspective of phenotypic plasticity (figure 1) [5,15–18]. We can think of organismal sex as the environment that alleles of a gene inhabit and within which they may get expressed [19,20]. Similarly, different cell types, tissues, organs and ontogenetic stages of development all can be viewed as distinct environments inhabited by an allele. An allele that confers differential expression of one or more genes across such environments, and that metes out a net positive influence on organismal fitness, represents an allele that confers adaptively plastic gene expression.

## 2. Evolutionary problems of adaptive plasticity

**Figure 3.**
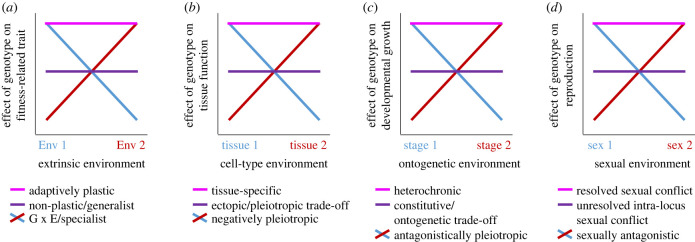
Tolerance curve representations of adaptive plasticity in distinct conceptual contexts. (*a*) Allelic effects on fitness-related traits in distinct environments can show patterns consistent with adaptive phenotypic plasticity (pink line), non-plastic generalist characteristics typical of a balance between the costs and benefits of experiencing different environments (purple line), or specialist effects such as local adaptation to just one environment that may indicate genotype-by-environment interactions (G × E; red and blue lines). (*b*) Adaptive plasticity of tissue-specific expression will enable multiple tissues to enjoy high functionality when expression may cause negatively pleiotropic effects. (*c*) Similarly, heterochronic gene expression across ontogeny represents a form of adaptive plasticity when constitutive expression leads to antagonistic pleiotropy across life stages. (*d*) Expression of a genotype that exerts sexually antagonistic effects can be resolved by the evolution of alleles with plastic expression that differs between the sexes, or partially resolved by alleles with constitutive expression at a level that balances costs and benefits between the sexes. Two ‘environments’ are depicted for simplicity in each panel, but multiple discrete expression contexts or a continuous range of contexts with nonlinear relationships may be considered in this same way.

When an organism's developmental trajectory responds to distinct environmental circumstances to alter a subsequent phenotype in a manner that enhances fitness in the corresponding environments, then the development of the trait demonstrates adaptive plasticity [9,21] (figure 3*a*). Classic examples include heterophyllic leaf shape of plants in aquatic and terrestrial growth, defensive spine production in *Daphnia*, and anhydrobiotic responses of tardigrades [22–24]. In these scenarios, cues from the external environment elicit alterations to the genetic networks that control developmental programmes to yield a different phenotype from an equivalent genotype. Here, I emphasize permanent developmental plasticity rather than reversibly plastic traits such as many of those involved in learning and behaviour [8,25]. Adaptively plastic phenotypes contrast with phenotypes that are highly robust to environmental perturbation, which instead show the same canalized developmental trait outcome irrespective of extrinsic conditions [26]; in such cases of canalization, the phenotype, rather than fitness, is robust to extrinsic conditions. Depending on the trait, such canalized phenotypes may or may not represent adaptive optima, and may or may not represent a fitness trade-off between competing selective pressures from distinct environments.

This view of environment-dependent trait development also applies to other developmental conflicts. First, expression of a given gene in one cell type may increase that tissue's functionality, whereas the same gene's expression in another cell type may depress that other tissue's functionality (e.g. ectopic expression of *Hox* genes [27]). Such antagonistic effects of an allele's expression in different contexts defines negative pleiotropy. Tissue-specific expression of the gene eliminates such negatively pleiotropic consequences and would permit each tissue to operate at maximum functionality to promote organismal fitness (figure 3*b*). Second, similarly, a gene's expression at different stages of development may impose opposing effects on proper developmental growth [28,29]. Temporally dynamic expression of such a gene to make it heterochronic would accommodate the ontogenetic trade-off associated with constitutive expression at a level suboptimal for both developmental stages (figure 3*c*). In species with haploid ‘gametophytic’ gene expression, haploid and diploid phases of the life cycle also may present ontogenetic environments with distinctive selective pressures [30–32]. In these ways, we may consider distinct tissue types or ontogenetic stages as analogues of distinct environments, for which adaptive plasticity as mediated through differential gene regulation can permit evolution of context-dependent fitness optima to resolve evolutionary conflict.

Finally, we can consider the sexual context within the body of an individual as an environment to which the development of a shared trait gets exposed [20,33,34]. Expression of autosomal genes provides a common currency for traits shared by the sexes. A gene's expression that influences the development of a sexual phenotype in one sex may improve the individual's reproductive output, whereas that same gene's expression in the other sex may depress the individual's reproductive fitness. This fitness trade-off in the development of shared traits between the sexes is one source of sexual conflict and sexually antagonistic selection, most easily considered in a mechanistic way for the case of intra-locus sexual conflict (figure 1) [35–37]. Sometimes this situation is referred to as a negative fitness covariance between the sexes due to cross-sex genetic correlations [38]. Constitutive expression of the gene would be expected to equilibrate at a level suboptimal for both sexes, a form of unresolved sexual conflict (figure 3*d*). Again, adaptive plasticity in gene expression will provide an evolutionary resolution [36,37,39,40]. Gene regulation that is adaptively plastic with respect to sex would permit the gene's expression to contribute to trait development in each sex to attain a value that maximizes the reproductive output of each individual of a given sex.

These links between tissue-specificity, heterochrony and sex-biased expression that all serve as developmental manifestations of adaptive plasticity may seem self-evident. Nonetheless, they correspond to an underappreciated shared basis to the negatively pleiotropic conflicts inherent to the context-dependence of trait states and gene expression. The measurement of gene expression provides a transferable data type among these domains, with analysis of gene regulation providing a unifying mechanism for exploring diverse aspects of plasticity of traits and the resolution of evolutionary conflicts. By highlighting these connections, lessons learned independently from each of the subfields of ecological adaptive plasticity, sexual conflict and evo-devo may prove mutually informative.

## 3. Genetic variation in the reaction norms of context-dependent traits

In addition to examining individuals that express a fixed allele of a given gene, we can consider genetic variation in the form of alternative alleles with distinct patterns of expression in distinct contexts [7,41]. That is, we can characterize the reaction norm of the development of a trait, or of the expression of a particular gene, separately for the effects of distinct genotypic forms. When the independent variable (*x*-axis in a reaction norm plot) is viewed as representing distinct environments, then distinct genotypic effects would be interpreted as a genotype-by-environment (G × E) interaction and could represent the basis to local adaptation by specialist genotypes (figures 1*b*, 2*b* and 3*a*). Countervailing selection pressures in distinct contexts provides one evolutionary means of maintaining genetic variation within a species.

We can also consider reaction norms and tolerance curves with respect to other circumstances in which a trait develops, beyond extrinsic environments (figure 3). When the ‘environments’ correspond to different developmental stages or tissues, then genetic variation in reaction norms and tolerance curves would represent alleles with negatively pleiotropic effects on the development of distinct stages or tissues (figure 3*b,c*). The possibility of such negative pleiotropy over ontogeny motivates the antagonistic pleiotropy model of ageing and senescence [29,42]. When viewed in terms of distinct sexual ‘environments,’ then we would interpret distinct genotypic effects as corresponding to sexually antagonistic alleles (figure 3*d*). In these circumstances, we may expect the maintenance of functional genetic variation with a suboptimal trait mean in the population that balances the trade-offs of expression and fitness in these distinct contexts [37,40,43–45].

We may then contrast these circumstances with predictions for what would happen should a new allele arise that permits differential expression in the different contexts. Such a new allele with context-dependent expression would ameliorate conflictual G × E interactions by permitting plasticity that enables higher fitness and function of a single genotype regardless of context. It would confer adaptive plasticity of gene expression and trait development, with evolution leading to trait states closer to the fitness optima defined by expression in different tissues, stages, sexes or extrinsic environments (figure 2). Consequently, we would expect the population genetic invasion of such an adaptive plasticity allele.

Do alleles exist that confer gene expression plasticity? Gene expression, like most traits, often shows extensive heritable variation within populations [46,47]. Moreover, like the extensive genetic variation for phenotypic plasticity in organismal traits [21,48], populations can indeed contain heritable variation for plasticity in gene expression [4,49,50]. Consequently, selection can act on such heritable variation to improve the alignment of fitness with expression plasticity. Some research traditions give special attention to the issue of genetic ‘accommodation’ or ‘assimilation’, associated with trait plasticity prior to genetic variation, in the course of trait evolution [51,52]. Regardless of the potential for ‘plasticity first’ perspectives, the existence of heritable variation in gene expression serves as the raw material for natural selection to drive the evolution of more (or less) pronounced plasticity [15,49,53–56]. It also is important to make clear that not all trait plasticity is adaptive [48]. We would not expect population genetic increase of alleles that confer plasticity in gene expression or trait development that do not simultaneously increase individual fitness.

**Figure 4.**
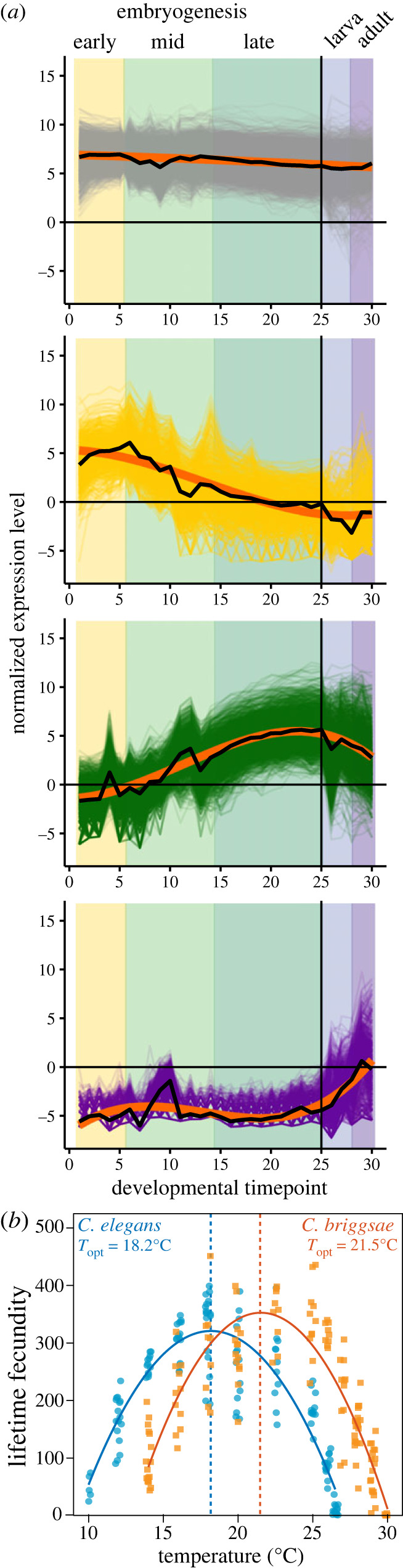
Phenotypic plasticity and tolerance curves represented as function-valued traits. For continuous dependent variables, including ontogenetic time as well as extrinsic environmental variables like temperature, an appropriate functional form may best summarize the trait plasticity or fitness relationship. (*a*) Gene expression dynamics across *Caenorhabditis elegans* ontogeny grouped genes into 14 coexpression profiles; four examples shown depicting the main trends of dynamic expression across development (constitutive, early-embryo peak, late-embryo/larval peak, adult-peak). Gene expression for each coexpression profile was then summarized by cubic polynomial functions (orange curves), with subsequent extraction of parameters for use as function-valued gene expression traits [60]. (*b*) Nonlinear thermal response curves for fecundity of *C. elegans* and *Caenorhabditis briggsae*, with *T*_opt_ inferred from the peak of quadratic function fits (data redrawn from [61]). These examples show function-valued traits inferred from ANOVA-style experimental design, but a regression-style experimental design is especially amenable to applying functional forms to summarize function-valued traits [62].

It also is important to consider the form of variation in the environment or expression context. The categorical nature of distinct tissues and the sexes naturally lends them to a discrete-state view of differential gene expression as captured with reaction norm analysis [25,57]. Adaptive plasticity in response to discrete extrinsic environments is well-appreciated, as for seasonal polyphenisms that can be tissue- and sex-biased in expression [58,59], and so tissue- and sex-specific expression more generally has ample analogues and precedent to analyse with such an approach [1,7]. For continuously varying ‘environments’, however, we can consider gene expression as a function-valued trait (figure 4) [62,63]. This more general approach that accommodates nonlinearity is well-suited to analysis of continuous extrinsic environmental variables such as temperature, as well as continuous features like the time course of ontogeny. For example, studies have used continuous functions to describe gene expression dynamics across ontogeny and then extracted parameter values as summary traits to more thoroughly capture the nature of gene expression than from any given snapshot during development [60,64,65].

## 4. Mechanisms of gene regulatory plasticity over developmental space, time and sex

To help link the principle of adaptive plasticity to gene expression plasticity in a mechanistic way, it is instructive to consider explicitly how mutation can create alleles that affect gene regulation to alter expression plasticity. Mechanistic studies provide ample specific examples of gene regulatory evolution that alters the context-dependence of gene expression over ontogeny, among tissues, between the sexes, and across extrinsic environments [3,66]. To frame the evolution of gene regulatory plasticity, I will first start with a scenario in which the ancestral state of a gene's expression is constitutive ubiquitous expression, a gene with an ‘open promoter.’ Such a gene could correspond to a ‘Class-A transcription factor,’ for example, in the terminology of Pope & Medzhitov [67].

One type of regulatory mutation could modulate the overall level of expression, increasing or decreasing it in all cells in a non-plastic way (figure 1*a*). For example, inducing constitutive hyperexpression of the sulfatase *eud-1* in *Pristionchus* nematodes leads to canalized production of one type of mouth form whereas eliminating expression of *eud-1* leads to canalized production of an alternative mouth form [68]. The kinds of regulatory mutations involved could result from either *cis*-acting local alterations near to the coding sequence that influence, e.g. nucleosome occupancy, or from *trans*-acting distant alterations that influence, e.g. motif binding strength [3,69]. Such a mutation could be adaptive relative to the ancestral allele. Specifically, this type of regulatory change would be responsible for the evolution of expression to a level that balances the trade-off between detrimental overexpression in one tissue, stage, sex or extrinsic environment with the detriments of underexpression in others (purple allele in figures 1–3). These sorts of alleles could also be considered to confer an intermediate ‘generalist’ expression phenotype. However, they would leave unresolved evolutionary conflict between competing fitness demands. Such competing demands—beneficial effects in some environmental contexts, detrimental effects in other contexts—represent a form of negative pleiotropy.

Another type of regulatory mutation could modulate the specificity of expression, increasing or decreasing it in just a subset of cells, stages, sex, or extrinsic environmental conditions. Such a mutation would create an allele with plasticity of expression. For example, 182 ‘expression plasticity quantitative trait loci’ were detected in *Caenorhabditis elegans* nematodes as genetically variable *trans*-acting gene regulators that showed temperature-dependent effects [50]. A *cis*-acting mutation, however, is perhaps the most obvious kind of such a change (pink allele in figure 1). It could serve as a co-activator/repressor or an inducible enhancer/silencer to promote differential expression of a single genotype either transcriptionally or post-transcriptionally, and changes to *cis*-regulatory elements (CRE) would tend to exert fewest additional pleiotropic effects [70–72]. Nonetheless, other possibilities include *cis*-acting splice modifiers of coding sequence to produce context-dependent alternative splice forms of proteins, *trans*-acting mutations that operate by regulating upstream transcription factors, or small RNA pathways and mutations that influence post-transcriptional regulation in context-dependent ways [18,36].

Different expression contexts may be prone to regulating expression through different pathways. For example, transcriptional regulation predominates for spermatogenesis genes whereas post-transcriptional regulation predominates for other germline genes in *C. elegans* [73]. Similarly, translational and post-translational regulators of proteins can mediate functional and developmental disparities among cells, stages, sexes and organisms in different extrinsic environments. Gene duplication, followed by regulatory divergence that confers subfunctionalization of expression, also provides an evolutionary pathway to resolution of conflicts over negative pleiotropy [74–76]. In the case of resolving negative pleiotropy over expression between the sexes in species with heteromorphic sex chromosomes, translocation of an autosomal gene to a sex chromosome can serve to restrict gene expression to one sexual environment [36]; analogously, resolution of tissue-specific pleiotropic conflicts (e.g. germline versus soma expression) might occur through gene translocation in species that undergo somatic chromosome diminution of portions of their genome [77]. Even coding sequence changes that influence only a portion of a protein's activity, owing to so-called differential pleiotropy of distinct protein domains [27], could mediate functional differences in different contexts. Any new mutation of this sort that permits differential gene expression or functional activity in a context-dependent way that enhances fitness would correspond to an allele conferring adaptive plasticity.

Epigenetic modifications can provide a proximate mechanism for plastic gene expression in ways that yield context-dependent effects [36,78,79]. Potential factors include methylation of DNA or histones, or other chromatin modifiers, as well as activity of some long non-coding RNA and small RNA pathways [80,81]. Here, I simply consider the evolution of alleles that control the upstream factors responsible for triggering any epigenetic activity or, alternately, evolution of alleles associated with a downstream gene that recruit epigenetic regulatory factors that could then provide the proximate source of modulation of a focal gene's expression.

What remains unresolved, however, is whether any of these molecular mechanisms represent paths of least resistance for the evolution of adaptive plasticity of gene expression as a resolution to evolutionary conflicts over negative pleiotropy in different contexts [20,82]. For example, plasticity in expression owing to *cis*-acting changes tend to accumulate in *Arabidopsis* to magnify pre-existing plasticity of expression [83]. Emerging studies that employ allele-specific expression to detect biased expression among a wide array of tissues and other contexts offer a potentially powerful means to address questions about different mechanisms of plastic gene regulation with respect to expression contexts [84,85]. In addition, it remains unresolved how often it is the case that observed context-dependent expression is, in fact, adaptive. Differential expression can arise through indirect selection on unrelated traits from genetic correlations in gene regulatory architectures, as shown for sexually dimorphic gene expression [86]. These issues make it valuable to test explicitly for links between dynamic gene expression, gene regulatory mechanisms and fitness.

## 5. Limits and facilitators of adaptive gene expression plasticity in the resolution of negative pleiotropy in development

We would generally expect the population genetic invasion of an ‘adaptive plasticity allele’ [7]. Such an allele need not provide optimal functionality in any one context, only that it confers highest organismal fitness in a weighted average across expression contexts relative to other alleles [87]. It would act to mitigate fitness conflicts by reducing the negatively pleiotropic effects of a suboptimal broad expression profile. If inducing sex-biased expression, in the jargon of sexual antagonism, such an allele would reduce or resolve sexual conflict. If inducing differential expression across ontogenetic stages, then such an allele would confer heterochronic expression to the gene. Heterochronic expression would promote the evolutionary resolution of negative pleiotropy across the life cycle (often referred to as antagonistic pleiotropy with respect to ageing and senescence [29,42]). If inducing differential expression among cell types, tissues or organs, then we would view the allele as conferring tissue specificity of expression that narrows the expression breadth of the gene. If inducing differential expression under distinct extrinsic environmental conditions to promote fitness, then the allele would correspond to traditional notions of adaptive phenotypic plasticity by conferring robustness to fitness in the face of environmental perturbations.

The evolution of adaptive plasticity arises more readily when environments get sampled in a ‘fine-grained’ way. The grain of selection is ‘fine-grained’ when organisms experience different environmental contexts reliably within each generation, in contrast to ‘coarse-grained’ sampling of environmental contexts over the timespan of different generations [88–92]. The grain of selection is equivalent to the notion of the frequency or predictability of exposure to a given selective environment, with important consequences for the evolution of adaptive plasticity [93,94]. A given gene's expression will experience the most consistent selection at the organismal level from ‘fine-grained’ environmental sampling across tissues and stages that occur within a generation (i.e. the grain of selection, *f*(*x*), approaches 1 in the formulation of [94]: *f*(*x*) → 1). An allele's expression in a sexual context has a more intermediate grain of selection than do tissues and stages, experiencing sex-specific selection only half of the time in a population with an even sex ratio (i.e. *f*(*x*) = 0.5 for autosomal loci, with sex linkage or duplication changing this value), and may encounter some extrinsic environments even more rarely (i.e. coarse-grained with *f*(*x*) → 0). Genes expressed late in adulthood also might be considered to have a coarser grain of selection than genes expressed early in life, owing to the diminishing reproductive value of individuals after reproductive maturity.

The probability of fixation of a new mutation that confers adaptively plastic gene expression is directly proportional to the grain of selection [93]. Consequently, adaptive plasticity in the form of context-dependent regulatory evolution should most easily evolve tissue-specificity, heterochrony and extrinsic environment-dependent expression for reliably encountered environments within a lifetime, followed by sex-biased expression and then extrinsic environment-dependent expression that arises from coarse-grained ecological heterogeneity. Extremely coarse-grained extrinsic environments with long intervals between encounters may provide insufficient selection to favour the evolution of plasticity, or may yield local adaptation instead, or may promote the maintenance of genetic variation in expression plasticity. Standing genetic variation as the basis to evolutionary conflicts thus ought to be rarest for tissue-biased and heterochronic expression. Moreover, interactions between different expression contexts can ‘coarsen’ the grain of selection [91,92]. Plastic expression of sex-biased traits can intersect with extrinsic environments, as for cases of evolution of within-lifetime and across-generation seasonal polyphenisms in sexual traits [58,59,95]. The effect size of G x E interaction for sexual traits is greater in coarse-grained extrinsic environmental contexts [91] and body condition-dependence as a manifestation of coarse-grained extrinsic environmental contexts may mask costs to plasticity [96]. The interaction between sex-biased expression with tissue- and stage-specific expression could also serve to make the grain of selection finer, however, potentially allowing easier evolutionary paths to resolution of some negative pleiotropies. The pervasive phenomenon of dynamic context-dependent gene regulation in development may thus reflect these evolutionary predispositions.

Any given regulatory mutation that is capable of conferring adaptive plasticity in gene expression need not fully resolve pleiotropic conflicts. It may simply represent one substitutional step towards that resolution on an adaptive walk (figure 2). Subsequent regulatory changes potentially may be capable of inducing greater and greater differential expression between tissues, stages, sexes or extrinsic environments. Consequently, any assumption about gene ancestry in terms of ubiquitous constitutive expression does not represent a necessary precondition for the evolution of new alleles to spread through a population by virtue of conferring adaptive plasticity.

What limitations to plasticity evolution are there, given that nature shows a preponderance of genes that show differential plastic expression across developmental space (cells, tissues), time (ontogenetic stage) and sex? Evolutionary theory nonetheless points to a variety of potential constraints on the evolution of adaptive plasticity [88]. One line of reasoning about evolutionary constraints holds that the capacity for plasticity itself may exact energetic or opportunity costs that limit its net benefit [48,97]. In the context of gene expression, plasticity will generally require more elaborate *cis*-regulatory architecture or more complex interactions among *trans*-acting factors that would provide a larger mutational target for deleterious mutations. In practice, however, these potential costs of such additional regulatory complexity may be negligible in most organisms [88,98].

A potential short-term constraint on the evolution of plasticity in gene expression as mediated by CRE polymorphisms is owing to CREs tending to be very short sequences. Consequently, any given CRE represents a small mutational target with low opportunity to generate new alleles with plasticity in expression [99]. This property may reflect the tendency for within-species genetic variation in gene expression to be comprised disproportionately of *trans*-acting variants [72,100]. The accumulation of regulatory divergence as fixed differences between species, however, tends to disproportionately incorporate *cis*-acting variants, reflecting the long-term introduction of mutations that get filtered by selection.

A possible indirect cost of regulatory complexity is a greater potential for catastrophic error, a form of developmental instability [48,97]. Such errors could plausibly arise under stressful conditions from perturbations of shared transcription factors, shared enhancers, or shared promoters of polycistronic genes (as found commonly in *C. elegans* nematodes), as molecular mediators of pleiotropy. It is less clear how important this hypothetical cost might prove to be in the context of gene expression plasticity among tissues, life stages and the sexes, but perhaps could be informed by theory on robustness and genetic network architecture [66,101,102].

Experimental evolution within stable extrinsic environments has provided one framework for quantifying costs of plasticity [103–107]. Costs of plasticity might also underlie the evolutionary loss of plasticity in gene expression and other traits during experimental evolution with directional selection, as observed in copepods [55]. Similarly, an experimental evolution paradigm of allelic competition [108,109], e.g. between transgenic engineering of constitutive versus context-dependent regulatory promoters, could help to quantify the costs and benefits of plasticity of expression among tissues, ontogenetic stages or sexes.

Even with minimal costs arising from regulatory evolution to confer adaptive plasticity in gene expression, the fitness benefits to further increases in plasticity will face diminishing returns. Another way of phrasing this idea is that the efficacy of selection on plasticity trades off with the extent of context-specific expression of a gene [93,110]. That is, as the fitness optimum of ‘perfect’ plasticity gets approached in an adaptive walk, the effect size of fitness gains will become more and more marginal (weaker selection). For example, the weaker force of late-life selection may enable the persistence of negative pleiotropy over ontogeny, as motivates the antagonistic pleiotropy model of ageing and senescence [29,42]. This weakening of selection is also sensitive to how much exposure to distinct conditions a given allele will experience (i.e. the grain of selection) [93,94]. Moreover, population size limitations on evolutionary responses may render effectively neutral any alleles that would confer more-refined adaptive plasticity (i.e. owing to ‘drift load’) [111]. Consequently, genetic variation for the degree of plasticity may persist within populations [66,93]. One way to explore such consequences of the evolution of expression plasticity is by quantifying accumulation of non-adaptive changes to coding sequences of genes with different degrees of context-dependent expression [110]. It is important to recognize in analyses of molecular evolution that the strength and form of selection on gene regulatory elements that control plasticity of expression can differ substantially from the strength and form of selection acting on the coding sequences of the genes that get expressed [112,113].

When considered from the perspective of extrinsic environmental heterogeneity, the evolution of adaptive phenotypic plasticity resolves conflicting selection pressures as a means of tolerance [2]. Consequently, plasticity leads to relaxed selection on context-specific developmental pathways and can permit the accumulation of cryptic genetic variation [88]. The underlying gene regulation will continue to evolve with the magnitude of differential context-dependent expression, and genetic variation in it, also potentially influenced by indirect selection on unrelated traits [86]. Interestingly, this idea may also extend to genes conferring adaptively plastic expression across tissues, stages and sexes. Reciprocally, developmental context can influence the efficacy of selection and therefore the likelihood that adaptive plasticity evolves.

For example, selection is less effective on genes with plastic sex-limited expression owing to the coarser grain of selection, which contributes to molecular evolutionary signatures of rapid evolution in genes with sex-biased expression [114–116]. Interestingly, the resolution of intra-locus sexual conflict through plastic expression also holds the potential to instigate inter-locus sexual conflict, with implications for subsequent arms-race evolution [36]. Gene expression before or after reproductive maturity also is expected to experience predictable differences in the force of selection [117], as considered in models of ageing and senescence [29,42], and of sexual selection [33,118]. More generally, coding sequences of genes with narrow expression breadth across tissues or ontogenetic time, owing to gene regulatory plasticity, tend to accumulate substitutions more readily owing to weaker negative selection and/or more positive selection [60,110,119–124]. These links suggest the potential for cross-pollination between ideas from evo-devo about cryptic genetic variation and developmental system drift, population genetic principles regarding selection efficacy, and ideas about sex-limited expression in sexual selection and sexual conflict.

In this discussion of allelic effects, I have neglected evolutionary conflicts that involve the influence of expression in one context affecting fitness experienced elsewhere. Inter-locus sexual antagonism provides perhaps the most familiar case, in which (plastic) sex-limited expression of an allele can lead to reduced fitness of the other sex as a result of inter-sexual interactions [35,125–127]. In principle, this idea can extend to other circumstances, such as tissue-specific expression in one organ leading to suboptimal function of another organ system within a given individual, though the fine grain of the selection regime will limit the possible evolutionary outcomes. Gene regulatory network modularity has also been implicated as a pertinent feature of a mechanistic multi-locus view of plasticity evolution [66], as context-dependent expression can arise as an indirect evolutionary response to other selection pressures [86]. Inter-locus antagonisms can drive allelic substitutions to accrue repeatedly in a ‘Sisyphean arms race’ that can involve mutations of larger effect than would be expected from adaptation to a fixed fitness optimum [125,127,128]. Perhaps such inter-locus arms race dynamics could foster the evolution of more baroque gene regulatory networks, with implications for network architecture to influence properties of modularity and robustness to perturbation.

## 6. Division of labour: an alternative view of the evolution of differential expression in development?

The evolution of specialization through the advantages to division of labour offers an alternative lens to view of the evolutionary benefits to differential expression. If the functional capacity of a cell type gives disproportionate fitness gains by virtue of the economies of scale from specialization, then we ought to expect the evolutionary outcome of tissue differentiation and organ specialization [129,130]. Such evolutionary division of labour in development is not restricted to organs within individuals, as it can contribute to specialization among castes of different individuals in some eusocial superorganisms, as well [131,132].

The appropriateness of applying a division of labour lens to the sexes, however, depends on the sexual system. In outcrossing anisogamous species with a single hermaphrodite sex, all individuals share the genome in its entirety with no sex-limited regions (i.e. entirely autosomal with no sex chromosomes). Similarly, the two sexes in haplodiploid organisms share the genome in its entirety [133]. Specialized expression of these cases offer straightforward extensions to the logic of division of labour. In hermaphrodites, division of labour for an ovotestis becomes a problem of sex allocation [134]. Simultaneously hermaphroditic organisms can access a continuous range of allocation strategies by virtue of producing both types of gametes concurrently [135,136], which can itself be viewed as a form of reproductive adaptive plasticity relative to separate sexes [137]. By contrast, reproductive allocation strategies in sequential hermaphrodites are discretized, making just one gamete type at a time [138]. Sequential hermaphroditism can be viewed as an evolutionary resolution to context-dependent reproductive fitness gain curves—disproportionate fitness gains with specialization—with respect to internal or external environmental conditions, as an adaptively plastic response [134,139]. Similarly, the development of separate sexes in species with environmental sex determination, of which sequential hermaphroditism is an exceptionally plastic form, may also be viewed as an analogue of tissue specialization. Viewing separate sexes of species that have genetic sex determination through the lens of division of labour, however, probably would require an inappropriate group selectionist outlook (some exceptions notwithstanding [140]). Consequently, the lens of adaptive plasticity appears more broadly unifying than does division of labour for considering differential expression across contexts.

## 7. Conclusion

Gene expression offers a common currency for understanding diverse problems of evolutionary conflict over negative pleiotropies. The regulatory evolution that affects gene expression as a trait helps to make clear the connections of alleles with negatively pleiotropic fitness effects in distinct contexts, and their evolutionary resolution through adaptive plasticity of gene expression. In particular, adaptive phenotypic plasticity in the form of gene regulatory evolution highlights commonalities among different kinds of evolutionary conflict involving antagonistic fitness effects of gene expression in different sexes, tissues, ontogenetic stages and extrinsic environments. The grain of selection is key to framing the most likely evolutionary response of alleles that confer adaptive plasticity of expression in these various contexts, and future work will be valuable that helps elucidate the potential costs of expression plasticity and whether particular gene regulatory mechanisms are predisposed to resolving some types of negative pleiotropies more than others. By recognizing the shared dependence of distinct research programmes on context-dependent gene regulatory evolution, and measurements of gene expression as a unifying data type, researchers from diverse fields may use the independently derived insights from one another.

## Data Availability

This article has no additional data.
